# Ecophysiological performance of terrestrial diatoms isolated from biocrusts of coastal sand dunes

**DOI:** 10.3389/fmicb.2023.1279151

**Published:** 2023-12-19

**Authors:** Karin Glaser, Sandra Kammann, Niklas Plag, Mirko Dressler

**Affiliations:** ^1^Institute for Biosciences, Biology/Ecology, TU Bergakademie Freiberg, Freiberg, Germany; ^2^Institute of Biological Sciences, Applied Ecology and Phycology, University of Rostock, Rostock, Germany; ^3^Department of Physical Geography, Institute for Geography and Geology, University of Greifswald, Greifswald, Germany

**Keywords:** light-irradiance curve, growth rate, desiccation, temperature extremes, stress tolerance, biocrust, terrestrial, sand dune

## Abstract

Terrestrial diatoms are widespread in a large variety of habitats and are regularly recorded in biocrusts. Although diatoms have long been known to live in terrestrial habitats, only a few studies have focused on their diversity of ecophysiology. Here we present a study on the ecophysiological performance of five terrestrial diatom cultures from biocrusts, which were collected in sand dunes of the German coast of the Baltic Sea. The sampling sites were selected along a gradient of human impacts on the dunes. The richness of diatom species, roughly estimated from permanent slides, was around 30 species per sampling site. The species abundance was calculated in the same way revealing a high proportion of broken diatom frustules. All diatom cultures established in the laboratory showed no photoinhibition and high oxygen production along a light gradient. The desiccation tolerance differed among the strains, with high recovery observed for *Hantzschia abundans* and *Achnanthes coarctata* and low to no recovery for *Pinnularia borealis* and *Pinnularia intermedia*. The maximum growth rate for most strains was between 25 and 30°C. These temperatures can be easily reached in their natural environments. Nevertheless, during short-term exposure to elevated temperatures, oxygen production was recorded up to 35°C. Interestingly, two of five diatom cultures (*Hantzschia abundans* and *Pinnularia borealis*) produced mycosporine-like amino acids. These UV-protective substances are known from marine diatoms but not previously reported in terrestrial diatoms.

## 1 Introduction

Sand dunes are the first geological formation along the sea on many natural shores. Coastal dunes are unique ecosystems in the transition zone between terrestrial and marine environments, where interactions between geomorphology, climate, and vegetation create highly dynamic environments (Martínez et al., 2004; Miller et al., 2010). However, dune ecosystems are threatened worldwide by human influences, such as land use and erosion (Hernández-Cordero et al., 2017). Coastal dune growth depends on sediment supply and stability, which are influenced by biotic (vegetation cover) and abiotic factors (wind, waves, and precipitation). Therefore, this ecosystem is vulnerable to declining sediment replenishment or surface degradation. Coastal dunes along public beaches are highly disturbed by trampling, mechanical cleaning, or permanent structures (e.g., groins for coastal protection) that impede wind-induced natural sand replenishment (Stancheva et al., 2011; Santoro et al., 2012).

Besides these human impacts on the coastal dune system, harsh environmental conditions with a variety of challenging environmental stressors such as strong winds, substrate mobility, nutrient, and soil water scarcity, occasionally extremely high near-surface temperatures, intense radiation, flooding, and salt spray (Maun, 2009; Miller et al., 2010) make the establishment of vascular plants quite challenging. Under these conditions, the growth and development of a closed vascular plant cover is restricted, except for anthropogenically planted marram grass (*Ammophila arenaria* (L.) Link). Under natural conditions, only specialized/stress-tolerant groups of organisms can establish on the dune surface; this includes cryptogamous communities such as biological soil crusts (biocrusts) (Schulz et al., 2016).

Biocrusts are formed by living organisms and their by-products, creating a topsoil layer of inorganic particles bound together by extrapolymeric organic compounds. Biocrusts are found on all continents, in arid, semiarid, and other habitats where soil moisture is limiting and cover of higher plants is sparse (Belnap et al., 2001). In temperate zones, these habitats include, for example, sandy coastal and inland dunes, disturbed areas (windbreaks, burned areas, etc.), or barren soils; biocrusts usually cover all soil areas not occupied by vascular plants and thus comprise up to 70% of the living cover (Belnap et al., 2001). Biocrusts form the most productive microbial biomass worldwide in the so-called ‘Earth’s Critical Zone’, which is the upper approx. 10 mm of soil in most dry areas. In ‘new’ vegetation-free or disturbed landscapes such as volcanic areas, glacier forelands, etc., biocrusts form the basis for further ecosystem development and succession (Eldridge and Tozer, 1996; Cutler et al., 2008; Yoshitake et al., 2010). Together with other microorganisms such as heterotrophic bacteria, archaea, and fungi, as well as macroscopic lichens and mosses, cyanobacteria and algae comprise the most important phototrophic components of biocrusts (Elbert et al., 2012). Because of their impact on various ecosystem functions, biocrusts can be characterized as ‘ecosystem engineers’. The cryptogam community forms water-stable aggregates that play important, multifunctional ecological roles in primary production, nutrient cycling, mineralization, water retention, soil stabilization, and dust binding (Evans and Johansen, 1999; Reynolds et al., 2001; Lewis, 2007; Castillo-Monroy et al., 2010). An overview of these microbiotic crusts clearly shows the important ecological role of these communities for global carbon (C) fixation (about 7% of terrestrial vegetation) and nitrogen (N) fixation (about 50% of terrestrial biological N fixation; Elbert et al., 2012).

Biocrusts in the temperate zone support a diverse algal community (Glaser et al., 2018; Mikhailyuk et al., 2019). Hundreds of different phototrophic species of cyanobacteria and algae (including diatoms) live in association with biocrusts. Terrestrial diatoms are widespread in many terrestrial habitats such as biocrusts, mosses, soil, caves, or artificial environments (Norbäck Ivarsson et al., 2013; Falasco et al., 2014; Kopalová et al., 2014; Schulz et al., 2016; Zhang et al., 2020). They contribute to soil stability by producing extrapolymeric substances, which function as glue for sand particles (Kidron, 2021). Furthermore, terrestrial diatoms are often reported even in high cell counts in biocrusts (Meadow and Zabinski, 2012; Schulz et al., 2016). However, their biodiversity, ecophysiology, and taxonomy are still inadequately characterized (Barragán et al., 2018). Marine diatoms are known to produce mycosporine-like amino acids (MAA) as UV-protective substances in response to increased solar radiation (Helbling et al., 1996; Jeffrey et al., 1999). In detail, there are solid indications that those MAAs are embedded in the silica frustule (Ingalls et al., 2010). MAAs were also recorded from terrestrial algae isolated from biocrusts, like for example *Mesotaenium* and *Klebsormidium* (Remias et al., 2012; Hartmann et al., 2020). It seems likely that terrestrial diatoms also follow this strategy and accumulate MAAs as UV-protective substances. Nevertheless, this fact has not been experimentally proven up to now. Only a few experimental ecophysiological studies on terrestrial diatoms have been undertaken under controlled conditions (Souffreau et al., 2010, 2013; Hejduková et al., 2019). These authors found similar response patterns for different terrestrial diatom species, as reflected in higher tolerances to extreme freezing and desiccation events that were lethal to isolates of freshwater diatom species. All other publications have provided comprehensive species lists and have attempted to find correlations between the occurrence or lack of terrestrial diatom taxa with key environmental factors (Foets et al., 2021). By applying an array of statistical methods (e.g., redundancy analysis), the preferential occurrence or lack of taxa can be explained by specific autecological requirements, such as pH or soil organic-matter content (Antonelli et al., 2017; Foets et al., 2021), yet experimental evidence is mostly missing. Nevertheless, taxa-specific indicator values for undisturbed and disturbed soil habitats, as well as tolerance ranges can at least be estimated from such correlations, and terrestrial diatoms are sensitive to multiple environmental factors such as pH, anthropogenic disturbance caused by farming practices (land-use intensity), and soil moisture and nitrogen contents (Foets et al., 2021). Farming practices play a key role in structuring soil diatom communities (Antonelli et al., 2017; Foets et al., 2021; and references therein), as disturbed areas were found to be less diverse and land uses with different disturbance levels could be differentiated based solely on the community composition. These authors also found that the composition of soil diatom species remained stable throughout the year and that diatoms were always present, in contrast to higher plants. Consequently, terrestrial diatoms could serve as indicator species in soils, similarly to the freshwater-diatom biotic indices used extensively to assess water quality. Recent studies have proven the suitability of diatom species assemblages as bioindicators for human disturbances, heavy-metal contamination, and soil environmental assessment (Wanner et al., 2020; Zhang et al., 2020; Minaoui et al., 2021).

In this study, we present a comprehensive ecophysiological characterization of five diatom cultures originating from biocrusts in coastal sand dunes, accompanied by an insight into the biodiversity of terrestrial diatoms in biocrusts, based on morphological identification. Our study aimed (1) to enhance our understanding of terrestrial diatoms by revealing unknown community structures of a yet-unstudied diatom habitat, the biocrusts in coastal sand dunes; and (2) to obtain insights into the ecophysiological performance of cultured terrestrial diatoms.

## 2 Materials and methods

### 2.1 Site description

**Figure 1:**
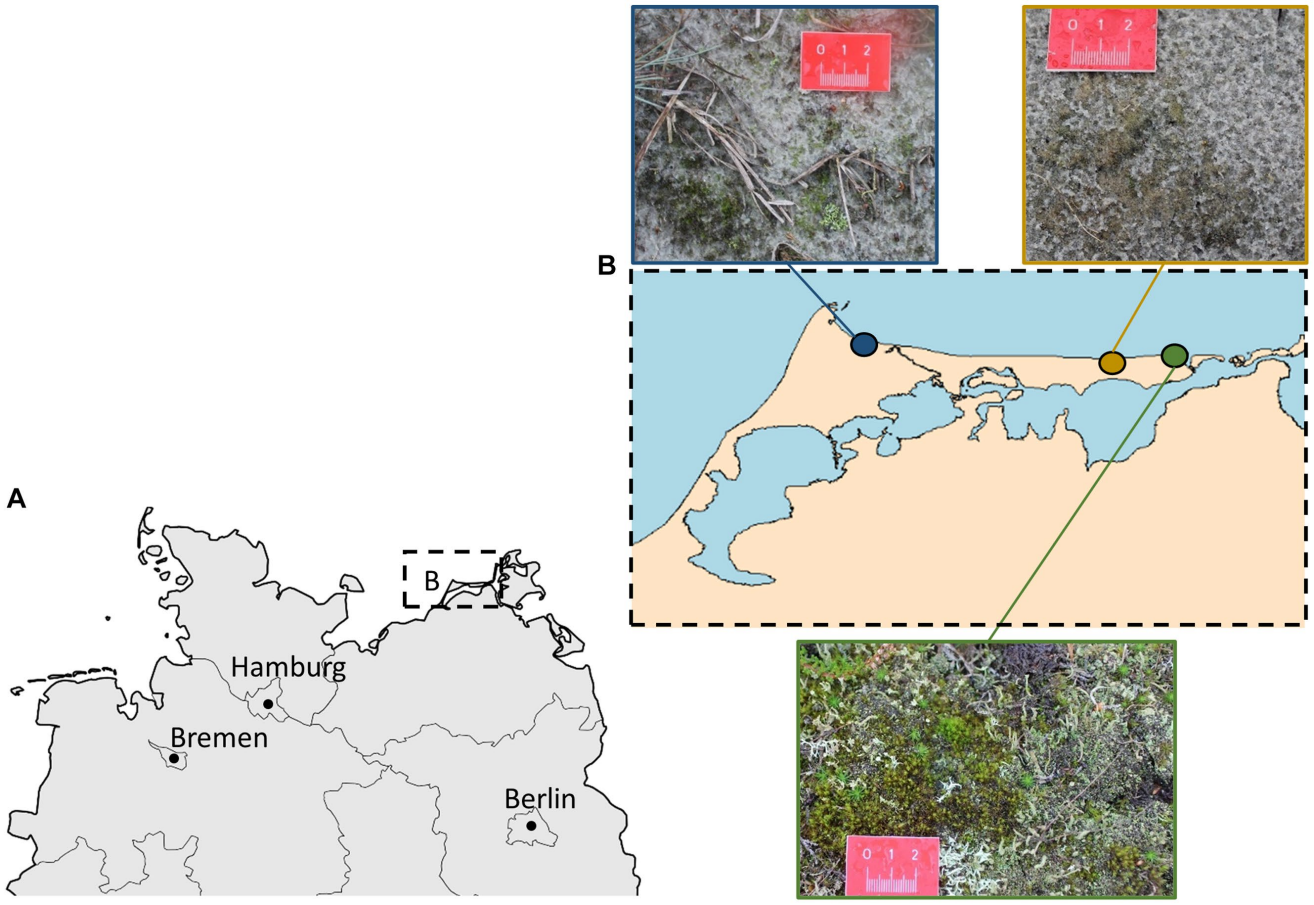
**(A)** The Darss-Zingst Peninsula in northern Germany. **(B)** Overview of sampling sites along the coastline of the Darss-Zingst-Peninsula, including impressions of biocrusts at these sites. Blue: camping area (highly disturbed); yellow: public beach (moderately disturbed); green: core zone of the National Park (little disturbed).

Biocrusts were collected on October 05, 2021 at three sampling sites along the coast of the Darss-Zingst-Peninsula, Germany (Figure 1). On that day, a temperature of 12°C and precipitation of 30.5 L m^−2^ were measured at the weather station in Zingst. The three sampling sites were selected according to their degree of disturbance by human activity: the highly disturbed site is located in a camping area (N54.45551, E12.54914); the moderately disturbed site is located at a public beach distant from the next village (N54.44103, E12.77921) in the care and development zone of the ‘Vorpommersche Boddenlandschaft’ National Park; and the little-disturbed site is located in the core zone of the national park (N54.44327, E12.90223). At the highly disturbed site, the dunes were clearly influenced by human trampling. Most of the area was bare sand and biocrust grew as thin biofilms between trampled paths. At the moderately disturbed site, trampled paths within the dunes were also visible, although it is forbidden to walk in the dunes. Biocrusts were visible as thin biofilm and were more frequently observed than at the highly disturbed site. At the little-disturbed site, no trampling was visible because a wooden boardwalk leads through the dunes. Here, the biocrusts were thicker, and even moss and lichen thalli could develop. A randomly chosen plot of 1 m^2^ was established at each of the three sampling sites for further material collection. Biocrusts were sampled by gently pushing a Petri dish into the surface and lifting it with a spatula. Five Petri dish samples were collected per site. These were sealed using laboratory sealing film and transported to the laboratory for further analysis.

The biocrust stability was measured in the field, using a penetrometer (fruit hardness tester FHT-15, tip width 3.5 mm diameter). The penetrometer was pushed at a 90° angle onto the biocrust surface until the biocrust broke. The measurement was repeated five times per sampling site.

In the laboratory, the chlorophyll *a* (Chl *a*) content of the biocrusts was measured as an estimator for photosynthetic biomass. A defined area of 0.5 cm^2^ biocrust was picked from the Petri dishes using a cork borer pushed approximately 1 cm into the sample. The biocrust material was incubated in 3 mL 96% ethanol at 78°C for 30 min. The solution was centrifuged and measured spectrophotometrically (Shimadzu UV-2401 PC, Kyoto, Japan) at 632, 649, 665, 696, and 750 nm absorbance. The last served as a control for turbidity (Ritchie, 2008). The extraction with ethanol was repeated until no chlorophyll *a* could be detected in the biocrust samples. Three replicates were measured for each sampling site. Total carbon and nitrogen (C_t_ and N_t_) contents were determined by dry combustion of about 100 mg ground biocrust sample material in an element analyzer (UNICUBE^®^ Elementar Analysensysteme GmbH, Langenselbold, Germany). Total phosphorus (P_t_) was extracted from 100 mg of ground material by sub-boiling digestion using an acidic persulfate solution in Teflon^®^ tubes for 24 h at 90°C (Berthold et al., 2015). All samples were neutralized using NaOH after digestion. The neutralized extract was further analyzed spectrometrically for phosphate, using the molybdenum blue method at 885 nm wavelength (Murphy and Riley, 1962). All total element contents were measured in five replicates per sampling site.

### 2.2 Preparation of permanent slides and morphological identification

Preparation of permanent slides followed the procedure described by Schulz et al. (2016). Briefly, approximately 0.5 g biocrust material was mixed with 4 mL distilled water and shaken. Immediately after shaking, 100 μL of overlying water was gently dripped onto glass coverslips, which were first air-dried and then combusted in a muffle oven (Elektra M26) at 550°C for 35 min. After cooling, the glass coverslips were mounted on glass microscope slides using Naphrax^®^. Diatom species were morphologically identified with the aid of a light microscope (Zeiss Axioplan, oil-immersion Plan-Apochromat objective, aperture 1.4) with 1,000-fold magnification. In total, over 400 valves that were at least 50% intact were counted per sampling site. The proportion of valves at least 90% and 100% intact was also recorded.

### 2.3 Establishment of diatom cultures

The establishment of terrestrial diatom cultures was challenging. The diatoms stuck tightly to the glass surface of the slides, making it impossible to pick single cells using a micromanipulator under a light microscope. Therefore, the pure cultures had to be developed by repeating transfer steps. Different culture media, both liquid and agarized, were tested to maximize the cultivation success: WC, Diat+Vit.mix (Bacillariophycean medium + vitamin mixture), BG11 (medium for cyanobacteria) and f/2 (enriched seawater medium) (recipes according to SAG). Initially, cultivation in agarized Petri dishes was more successful. After several transfer steps, only five diatom strains remained. They were transferred to liquid media where they could be established as unialgal cultures. These unialgal diatom cultures were cultivated in a Diat+Vit.mix medium at 20°C under low light (50 μmol photons m^−2^ s^−1^).

### 2.4 PCR and sequencing for identification

DNA was extracted from the five diatom cultures, following the instructions of the NucleoSpin^®^ Plant II Mini Kit (Macherey and Nagel, Düren, Germany). For identification, the rbcL gene (RuBisCO large subunit) was amplified in a PCR using a commercial PCR-Mastermix (Bioline). The primers Diat-rbcl-iR and Diat-rbcl-F were applied with the respective PCR protocol (Abarca et al., 2014). Sequencing was carried out by a commercial company (Eurofins, Luxembourg), using the same forward primer as for PCR. The sequences were uploaded to NCBI under the accession numbers OR387857–OR387860.

### 2.5 Desiccation experiment

The experiment followed the procedure described by Karsten et al. (2016). Briefly, the diatom cultures were grown for 1 week on glass-fiber filters (five replicates per strain), which were transferred for the experiment to a desiccation chamber filled with 100 mL silica gel. These were kept at a room temperature of ~25°C. The yield of photosystem II (YII) was recorded during desiccation every 10 min for 4 h as a proxy for the fitness of the cells, using non-invasive pulse amplitude modulation fluorometry (PAM2500, Walz, Germany). After a signal could no longer be measured, the filters were rewetted with 250 μL medium and transferred to another water-saturated chamber. The relative humidity in the chamber was continuously recorded in each second, using a multifunctional data logger (MSR 145 W; MSR Electronics GmbH, Switzerland).

### 2.6 Photosynthesis-irradiance curves

Photosynthesis-irradiance (PI)-curves of the five diatom strains (four replicates per strain) were measured according to Prelle et al. (2019). Briefly, 3.1 mL of thin log phase algal suspension of each strain and 31 μL sodium bicarbonate (NaHCO_3_, 2 mM final concentration) were added to four airtight water-tempered (20°C) oxygen electrode chambers (DW1, Hansatech Instruments, King’s Lynn, United Kingdom). The oxygen concentration was measured at ten increasing photon flux density levels ranging from 0 to ~1.500 μmol photons m^−2^ s^−1^ of photosynthetically active radiation (PAR), using a non-invasive oxygen dipping probe (DP sensors PreSens Precision Sensing GmbH, Regensburg, Germany). Measurements consisted of a 30 min respiration (dark) phase, followed by a 10 min photosynthesis (light) phase for each light level. The first and last minutes of each phase were excluded from the calculation. After the last measurement, Chl *a* was extracted from the 3.1 mL algal suspension (10 mL, 96% ethanol at 70°C for 10 min) and quantified spectrophotometrically (Ritchie, 2008). The mathematical photosynthesis model of Walsby (1997) was used for fitting and calculation of the maximum rates of net primary production (NPP_max_), respiration (R), light utilization coefficient (α), photoinhibition coefficient (β), light saturation point (I_k_), and the light compensation point (I_c_).

### 2.7 Temperature curve

The photosynthetic and respiratory response of each strain (four replicates per strain) at temperatures between 5°C and 40°C was measured using the same oxygen optode system as for the PI-curves (Karsten et al., 2010). After 20 min incubation in the dark, the respiratory oxygen consumption (10 min in the dark), followed by the photosynthetic oxygen production (10 min under light-saturated conditions at 335 μmol photons m^−2^ s^−1^ PAR) were determined. Measurements were normalized to the total Chl *a* concentration (see procedure above). The model of Yan and Hunt (1999) was used to fit the temperature values, including optimum and maximum temperature.

### 2.8 Growth rate

The fluorescence of Chl *a* was used as a proxy for biomass to calculate the growth rates of the five diatom strains according to the temperature. The *in-vivo* Chl *a* fluorescence measurements were performed with a self-constructed growth fluorimeter based on the basic electronic unit of an MFMS fluorimeter (Hansatech Instruments, King’s Lynn, United Kingdom) according to the protocol of Karsten et al. (1996). Bright-blue light LED emission (Nichia, Nürnberg, Germany) with a peak emission wavelength of 470 nm was selected for excitation of the Chl *a* fluorescence and pulsed with a modulation frequency of 870 Hz. Chl *a* fluorescence was detected as relative units by an amplified photodiode and was separated from scattered excitation light through a long-pass glass filter (RG 665; Schott, Mainz, Germany) and a bright-red gelatin filter (Lee, Brussels, Belgium). *In-vivo* Chl *a* fluorescence units correlate well to the cell number and the concentrations of organic carbon and Chl *a* in diatoms, as shown by Karsten et al. (1996) and Gustavs et al. (2009). The cultures were grown in disposable plastic Petri dishes with cover lids, in a volume of 20 mL culture medium, and measured every 24 h for 10 days, following the procedure of Gustavs et al. (2009). The light was kept constant at 45–105 μmol photons m^−2^ s^−1^, following a 16:8 light:dark cycle (Lumilux Deluxe Daylight L15W/950; OSRAM). The cultures were kept in water baths or air-conditioned rooms to ensure constant temperature conditions at five tested temperatures (5, 15, 20, 25, and 30°C), and all diatom strains were measured in triplicate. To ensure constant temperature over the course of the experiment, the light intensities were a bit below saturated conditions, because the light bulbs would otherwise have caused unwanted temperature fluctuations. Growth rates were calculated separately for each replicate, using the phase where the fluorescence signal increased exponentially (Gustavs et al., 2009). For 30°C, no growth rate could be calculated, because decreasing signal intensities were measured during the duration of the experiment. The model of Yan and Hunt (1999) was used to fit the temperature values, including optimum and maximum temperatures.

### 2.9 Detection of biochemical UV-protective substances

The biocrust biomass was extracted and further processed for HPLC (High-performance liquid chromatography) analysis as described by Karsten et al. (2009). Samples were analyzed with an Agilent HPLC system (Agilent, Waldbronn, Germany), and mycosporine-like amino acids (MAAs) were separated on a Phenomenex Synergi Fusion RP-18 column (Reversed Phase, Polar embedded C18 with TMS endcapping; 4 μm, 250 × 3.0 mm I.D.) protected with an RP-18 guard cartridge (20 × 4 mm I.D., Phenomenex, Aschaffenburg, Germany). The mobile phase contained 2.5% methanol (v/v) in 0.1% acetic acid (v/v) in HPLC water (0.055 μS cm^−1^) and was run isocratically at a flow rate of 0.5 mL min^−1^ at 30°C column temperature for 20 min. MAAs were detected with a photodiode array detector at 330 nm wavelength, and absorption spectra (290–400 nm) were recorded in each second, directly on the HPLC-separated peaks. MAA standards were run within the sample sequence. Those included Asterina-330 (retention time 3.33 min, absorbance at 330 nm), Shinorine (4.71 min, 334 nm), Prasiolin (9.6 min, 323 nm), Porphyra-334 (5.89 min, 334 nm), Mycosporine-glycine (3.8 min, 310 nm), Klebsormidin A (5.35 min; 323 nm), and Klebsormidin B (5.74 min; 323 nm).

### 2.10 Statistical analyses

All statistical analyses were done in R, version 4.2.1 (R Development Core Team, 2022) or Microsoft Excel. Significant differences between measured soil parameters for the biocrusts of different sites were calculated in R, using one-way ANOVA. Photosynthetic irradiance curves were fitted using the Walsby model in Excel, based on least-square methods (Walsby, 1997). Temperature curves (both growth rate and oxygen production) were fitted using the Yan and Hunt (1999) model in R, also based on the least-square model. The model by Yan and Hunt is a simplified exponential and polynomial model, which represents the plant growth below and above the temperature optimum better than bi-or multilinear models. It has proven to fit the experimental growth data of microalgae well (Losa et al., 2020). Significant differences between oxygen production and consumption along the temperature gradient were calculated using one-way ANOVA followed by a *post-hoc* Tukey test (*p* < 0.05). Confidence intervals for maximum oxygen production, maximum growth rate, and optimum and maximum temperatures were calculated using the command ‘confint2’ (package nlstools; Baty et al., 2015).

## 3 Results

### 3.1 Analyses of biocrust characteristics

The Chl *a* content of the little-disturbed site was much higher (189.17 mg m^−2^) than for the moderately and highly disturbed sites (72.32 and 76.62 mg m^−2^ respectively; Table 1). Further, the biocrust at the little-disturbed site was significantly more stable (1.12 MPa) than at the other two sites (0.5 MPa at the moderately and 0.4 MPa at the highly disturbed site). The stability of the biocrusts differed significantly between the little-disturbed and the other two sites (*p* < 0.05). The concentrations of total N (3.68 g  kg^−1^) and total C (78.76 g  kg^−1^) were significantly higher (*p* < 0.05) at the little-disturbed site. In contrast, significantly higher (*p* < 0.05) total P concentrations were measured in the biocrust samples from the moderately disturbed sampling site (50.7 mg  kg^−1^).

**Table 1:** Biocrust characterization of all three sampling sites.

Study area	Degree of disturbance	C_t_ (g kg^−1^)	N_t_ (g kg^−1^)	P_t_ (mg kg^−1^)	Chl *a* (mg m^−2^)	Stability (MPa)
Camping area	High	9.5 ± 3.6	0.5 ± 0.2	6.8 ± 4.4	76.6 ± 29.0	0.4 ± 0.2
Public beach	Moderate	1.5 ± 0.3	0.2 ± 0	50.7 ± 19.8	72.3 ± 49.9	0.5 ± 0.1
National Park	Low	78.8 ± 23.7	3.7 ± 1.1	19.4 ± 9.4	189.2 ± 24.3	1.1 ± 0.1

Means ± standard deviations (stability, C_t_ = total carbon, N_t_ = total nitrogen, P_t_ = total phosphorus, *n* = 5; Chl *a n* = 3).

### 3.2 Diatom species and relative abundance in biocrusts

In total, we observed 47 diatom species in biocrusts from three sand dunes on permanent slides (Supplementary Table S1). Each site showed a similar richness (28–30 species), all diatom taxa were pennate species. Six of the 47 diatom species could be assigned to a pure terrestrial life cycle, according to the literature. These were *Pinnularia borealis*, *Pinnularia. intermedia*, *Hantzschia amphioxys*, *Luticola terrestris, Mayamaea fossalis*, and *Nitzschia cf. pusilla*. These six diatom species were abundant in all the biocrust samples: 26% in biocrusts from the national park, 50% in biocrusts from the moderately disturbed area, and 18% in the highly disturbed dune area (Supplementary Table S1). The Simpson diversity index for the moderately disturbed site was lower (0.77) than at the other two sites (0.91 and 0.92). This was mainly because one species, *P. intermedia*, dominated the community at the moderately disturbed site, with an abundance of 45%.

Notably, many diatom frustules were partly disrupted. At the little-and moderately disturbed sites, approximately 48% were at least 90% or 100% intact. At the high disturbed site, only 25% were at least 90% or 100% intact (more details on the exact numbers of totally/mostly intact frustules can be found in Supplementary Table S1).

**Figure 2:**
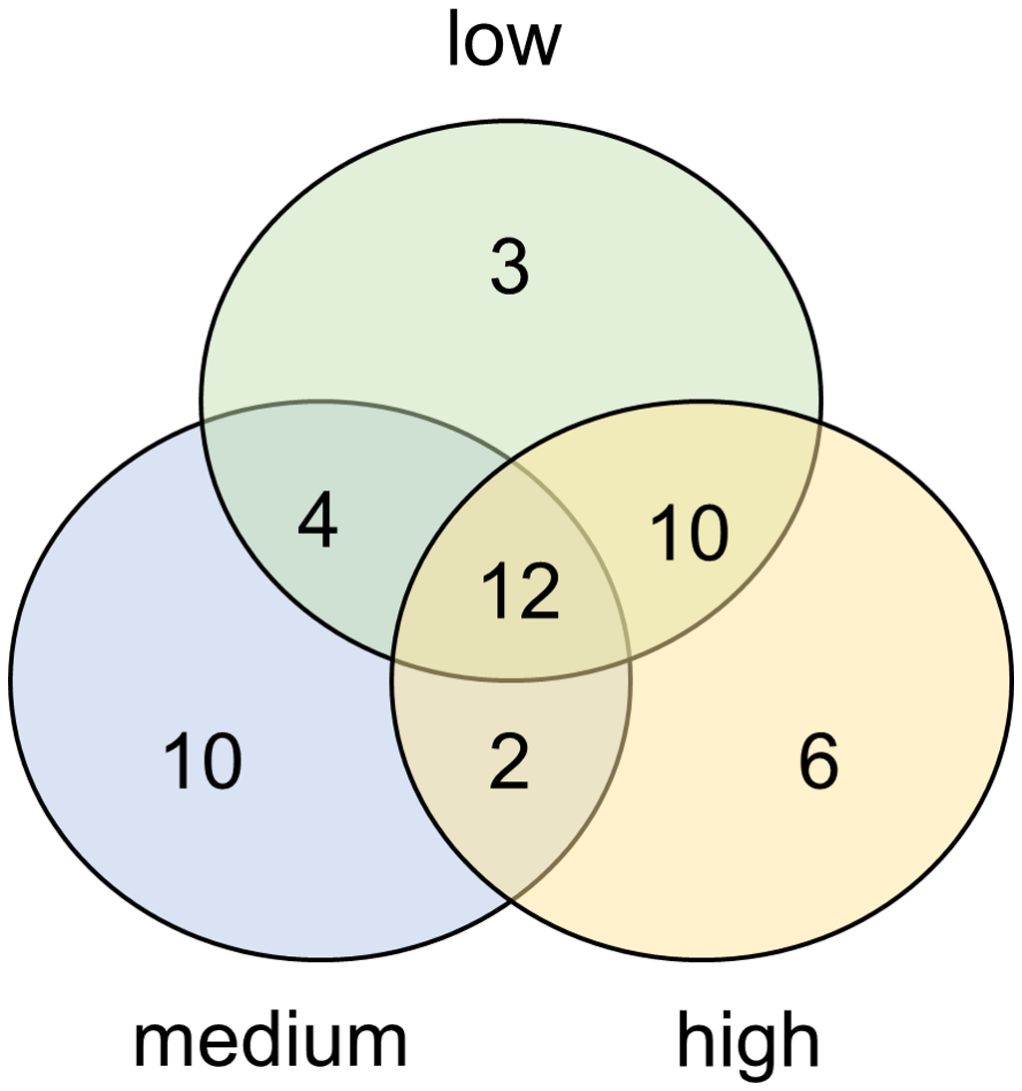
Venn diagram displaying the overlapping and unique diatom species present in biocrusts from dunes with high, moderate, or low degrees of human disturbance. Results based on morphological determinations from permanent slides.

Most of the diatom species were found at all three sites (Figure 2) but some (~38%) were found exclusively at one site. These unique species were in most cases scarce, whereas the common species for all three sites were highly abundant.

### 3.3 Identification of diatom cultures

We successfully developed five unialgal cultures of terrestrial diatoms from the sand dune biocrusts. Unfortunately, one culture collapsed during the experiments. This culture (*P. intermedia*) had relatively small cells (smaller than average according to the literature) and those cells became smaller and smaller over time until the culture was no longer viable. This phenomenon is known to occur in some diatom cultures. With each cycle of asexual reproduction, the average size of diatoms in a culture decreases due to the formation of silica frustules, until the species’ physical limit is reached. In the case of sexual reproduction, the daughter cells can become larger than the parent cells. However, not all diatoms reproduce sexually under culture conditions. Most likely, our culture collapsed because it reached its lower cell size limit and could not reproduce sexually under our culture conditions. Diatoms in the other cultures did not decrease in average size.

**Figure 3:**
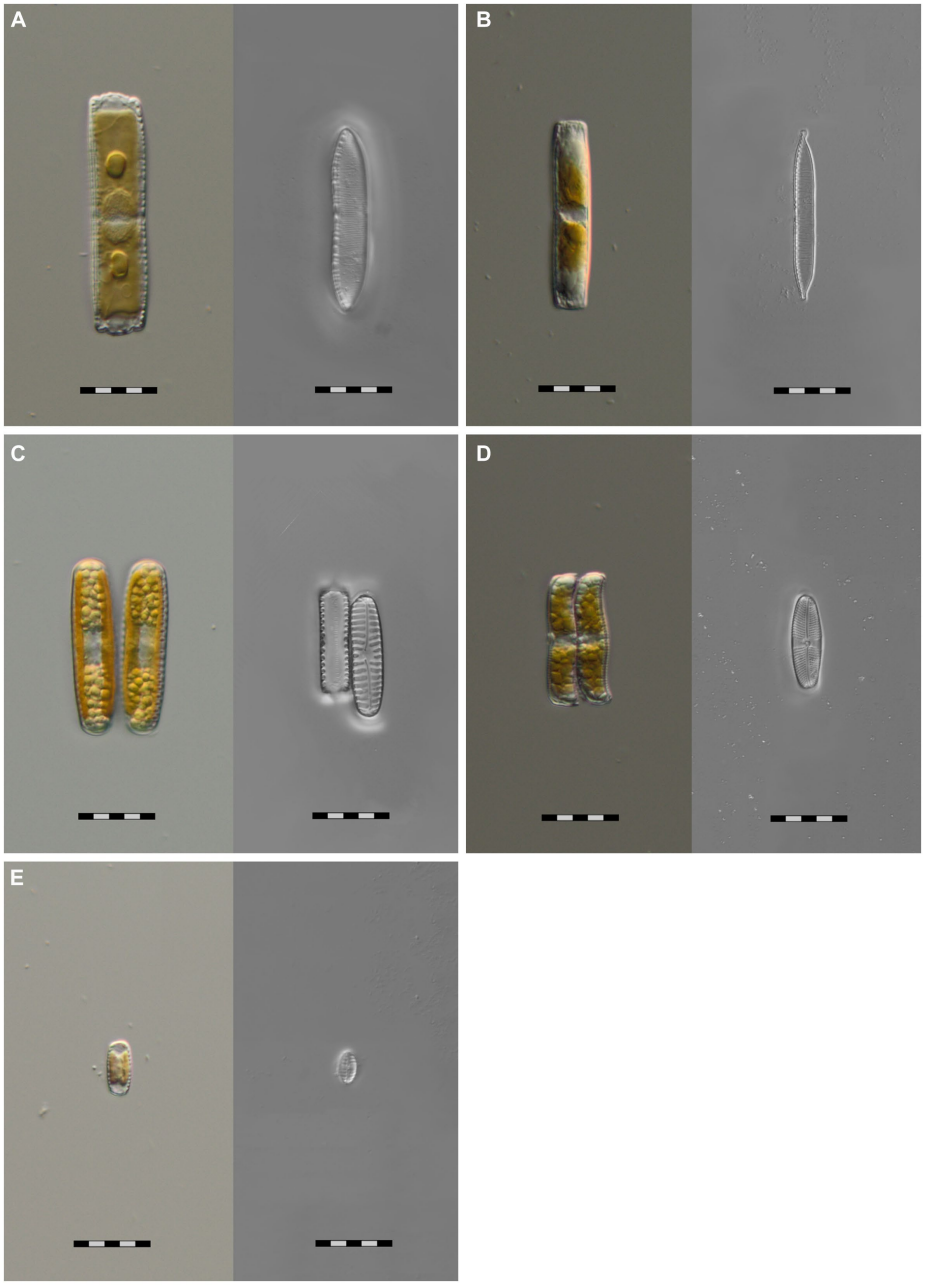
Microphotographs of five unialgal living diatom cultures and their frustules, isolated from biocrusts in coastal sand dunes, Mecklenburg-Vorpommern, Germany. Species identification is based on morphological and molecular features. **(A)** *Hantzschia abundans*, **(B)** *Hantzschia amphioxys*, **(C)** *Pinnularia borealis*, **(D)** *Achnanthes coarctata*, **(E)** *Pinnularia intermedia*; scale bar = 20 μm.

We successfully cultivated *Hantzschia amphioxys*-aggr. (#1), *Achnanthes coarctata* (#2), *Hantzschia abundans* (#3), *Pinnularia intermedia* (#4), and *Pinnularia borealis*-aggr. (#6) (Figure 3)*. H. amphioxys* and *P. borealis* originated from the highly disturbed site, whereas the other three cultures originated from the moderately disturbed site. The cultures were identified morphologically and also at the molecular level, based on the rbcL gene. The rbcL sequence of *P. borealis* was very similar (99.8% similarity) to the strain TAS17-48-10, which was assigned to clade 3 within the *Pinnularia borealis*-complex (Pinseel et al., 2019). The closest hit for the cultured *P. intermedia* was *P. acrosphaeria*, with 97.7% identity. There are no records of *P. intermedia* in the NCBI databases.

### 3.4 Desiccation tolerance

**Figure 4:**
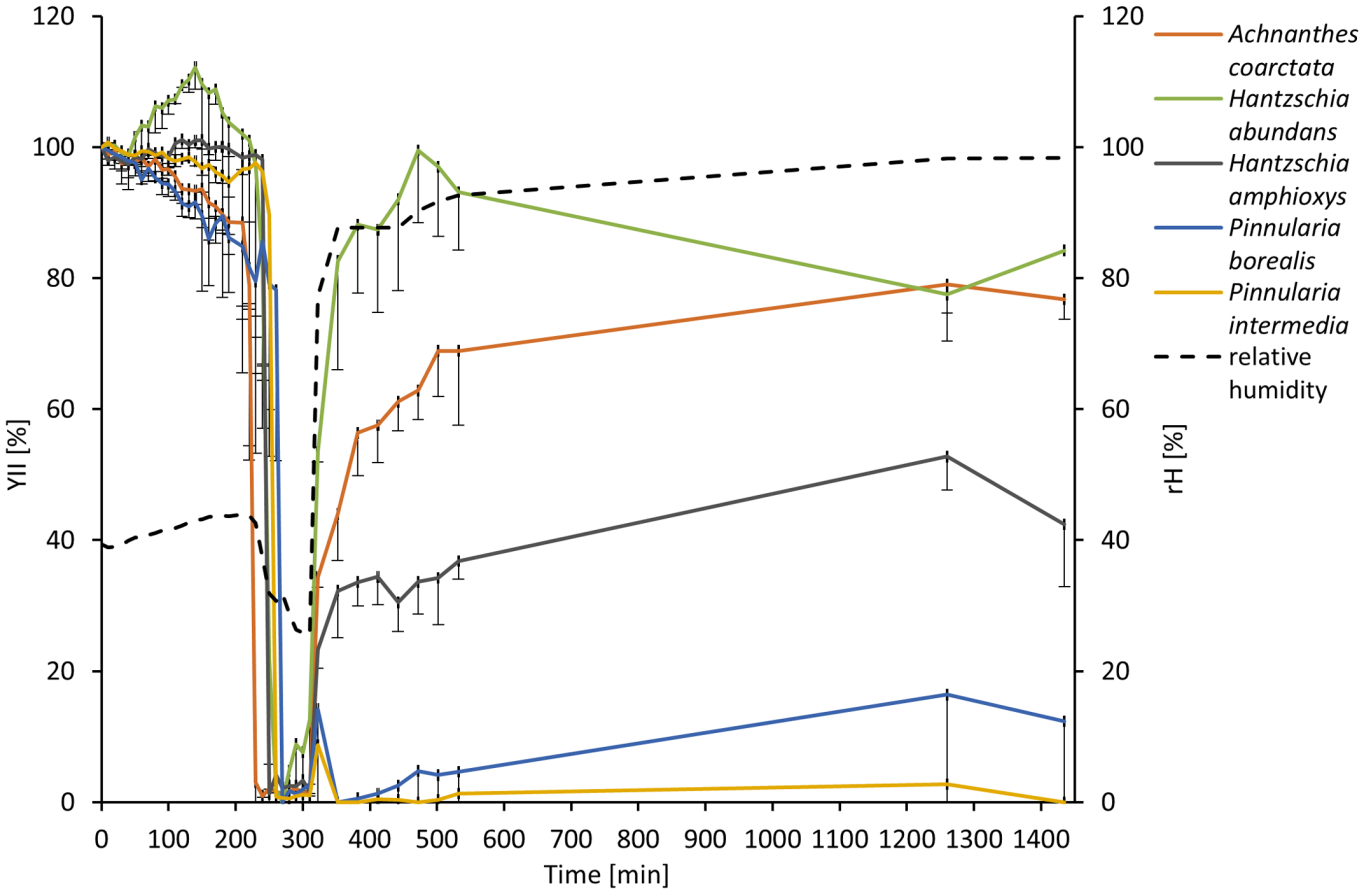
Effect of controlled desiccation and rehydration on the effective quantum yield (Y(II)) of PSII to five diatom cultures isolated from biocrusts in sand dunes (*n* = 4, only negative standard deviation is displayed for a better overview). Effective quantum yield values were standardized to the starting Y(II) to 100% for better comparison. The dashed line represents the measurement of relative humidity in the desiccation chamber.

The diatom cultures were subjected to desiccating conditions to estimate their desiccation tolerance. After around 4 h, the filters with diatom cultures dried completely in the desiccation chamber (Figure 4). The drop in relative humidity from 40% to ~25% was accompanied by a drop in the yield of photosystem II (YII) to 0%. Few minutes later, the filters were transferred to a water-saturated chamber. *P. intermedia* and *P. borealis* showed only minor recovery to the initial Y(II) after rewetting. *H. amphioxys* recovered to around 40% of its initial Y(II). *H. abundans* and *A. coarctata* performed best, ending with 80% of the initial Y(II) after 24 h (Figure 4).

### 3.5 Light-dependent photosynthesis

**Figure 5:**
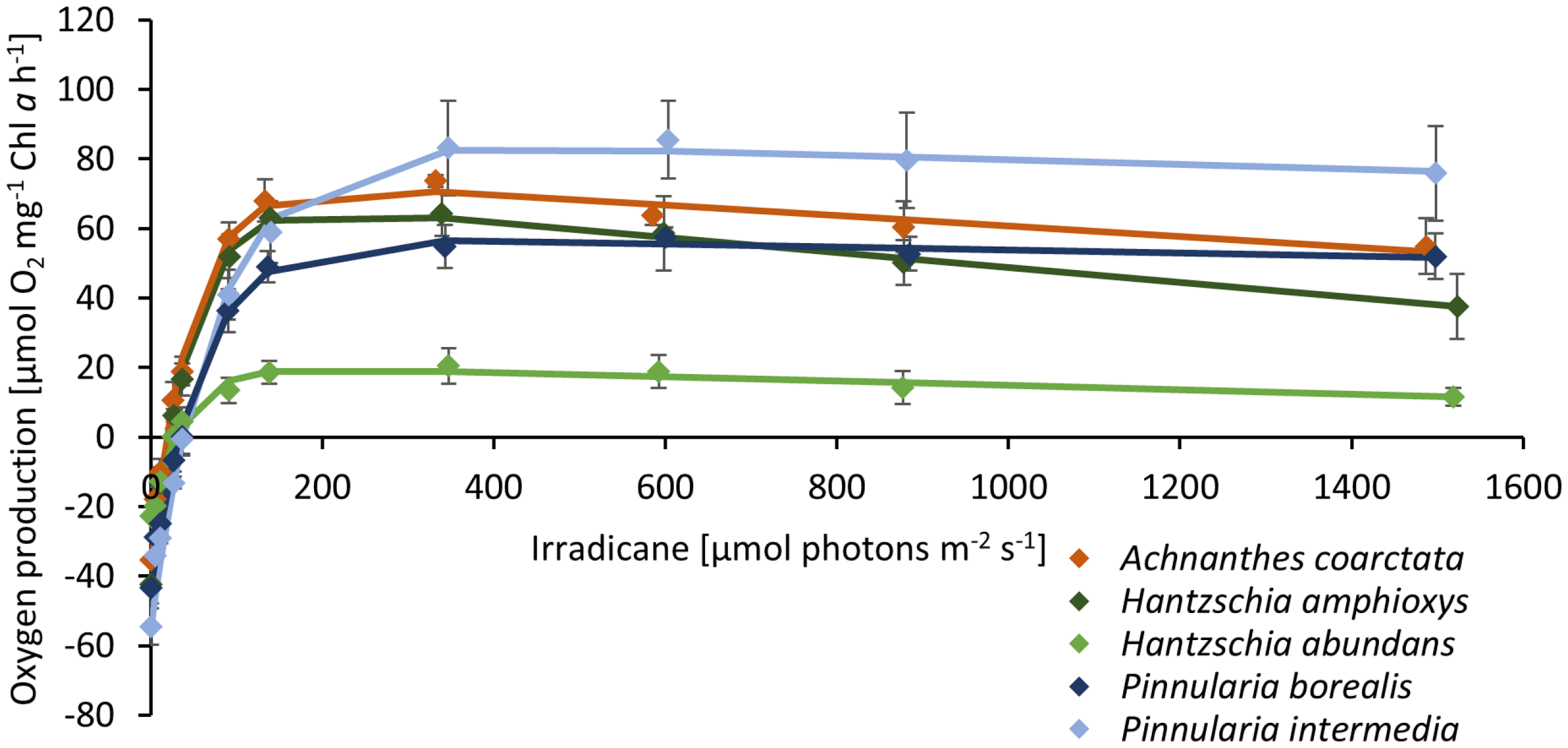
Photosynthetic-irradiance curve of five diatom cultures isolated from biocrusts in sand dunes. The points represent the mean of the measured values (*n* = 4 ± standard deviation), and the line is the fitting curve after Walsby (1997).

The photosynthetic performance of the diatom cultures was measured as oxygen production along an increasing light gradient. *H. abundans* had a lower NPP_max_, lower alpha, and lower respiration rate compared to the other four cultures (Figure 5; Table 2). All cultures showed no to only slight photoinhibition up to an irradiance of 1,500 μmol photons m^2^ s^−1^.

**Table 2:** Parameters of photosynthesis-irradiance (PI)-curves (Figure 4) of the five terrestrial diatom cultures (*n* = 4 ± standard deviation) measured at 20°C in Diat+Vit.mix medium.

	*Achnanthes coarctata*	*Hantzschia abundans*	*Hantzschia amphioxys*	*Pinnularia borealis*	*Pinnularia intermedia*
NPP_max_	71.27 ± 1.4	19.6 ± 4	64.93 ± 5.1	56.42 ± 3.7	82.84 ± 10.8
alpha	2.3 ± 0.5	1.11 ± 0.9	2.5 ± 0.1	1.72 ± 0.1	1.85 ± 0.2
beta	−0.01 ± 0.01	−0.01 ± 0.004	−0.02 ± 0	0 ± 0.004	−0.01 ± 0.01
Respiration	−35.42 ± 2.5	−22.62 ± 1.2	−42.3 ± 1.6	−43.24 ± 4	−54.56 ± 4.5
I_k_	46.29 ± 12.8	38.17 ± 8.9	42.91 ± 8.3	57.96 ± 12.3	74.32 ± 12.1
I_c_	18.68 ± 2.2	29.13 ± 4.3	21.51 ± 1.1	32.91 ± 1.6	37.56 ± 2.5

NPP_max_, maximum rates of net primary production in μmol O_2_ mg^−1^ Chl *a* h^−1^; alpha, light utilization coefficient; beta, photoinhibition coefficient; I_k_, light saturation point; I_c_, light compensation point.

### 3.6 Temperature-dependent photosynthesis

**Figure 6:**
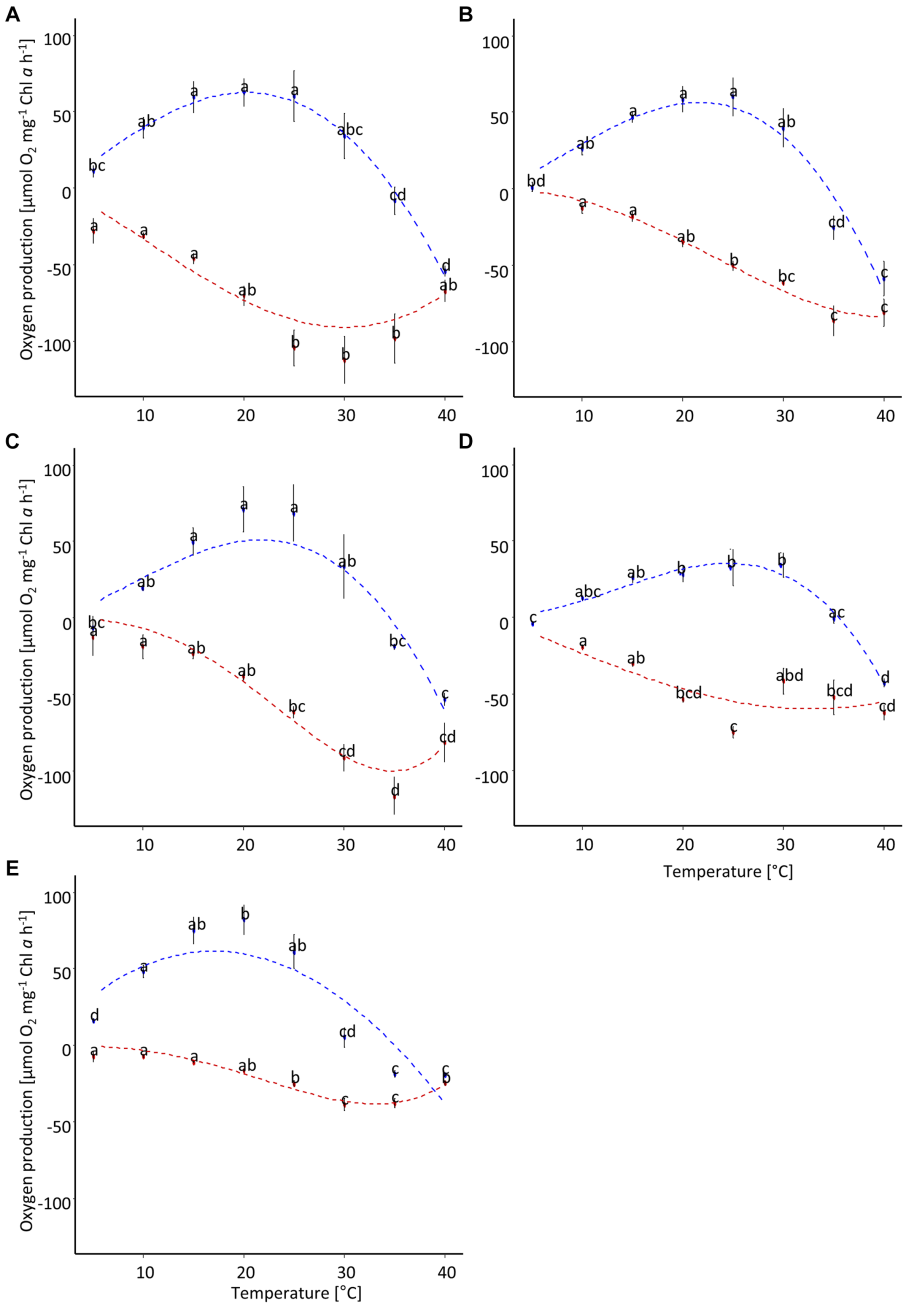
Temperature-dependent oxygen production of five diatom cultures isolated from biocrusts in sand dunes. The points represent the mean of the measured values (*n* = 4 ± standard deviation), and the dotted line is the fitting curve after Yan and Hunt (1999). Different letters indicate significant differences for photosynthesis (blue) or respiration (red), respectively (ANOVA with *post-hoc* Tukey test). **(A)** *Achnanthes coarctata*, **(B)** *Hantzschia abundans*, **(C)** *Hantzschia amphioxys*, **(D)** *Pinnularia intermedia*, **(E)** *Pinnularia borealis.*

**Figure 7:**
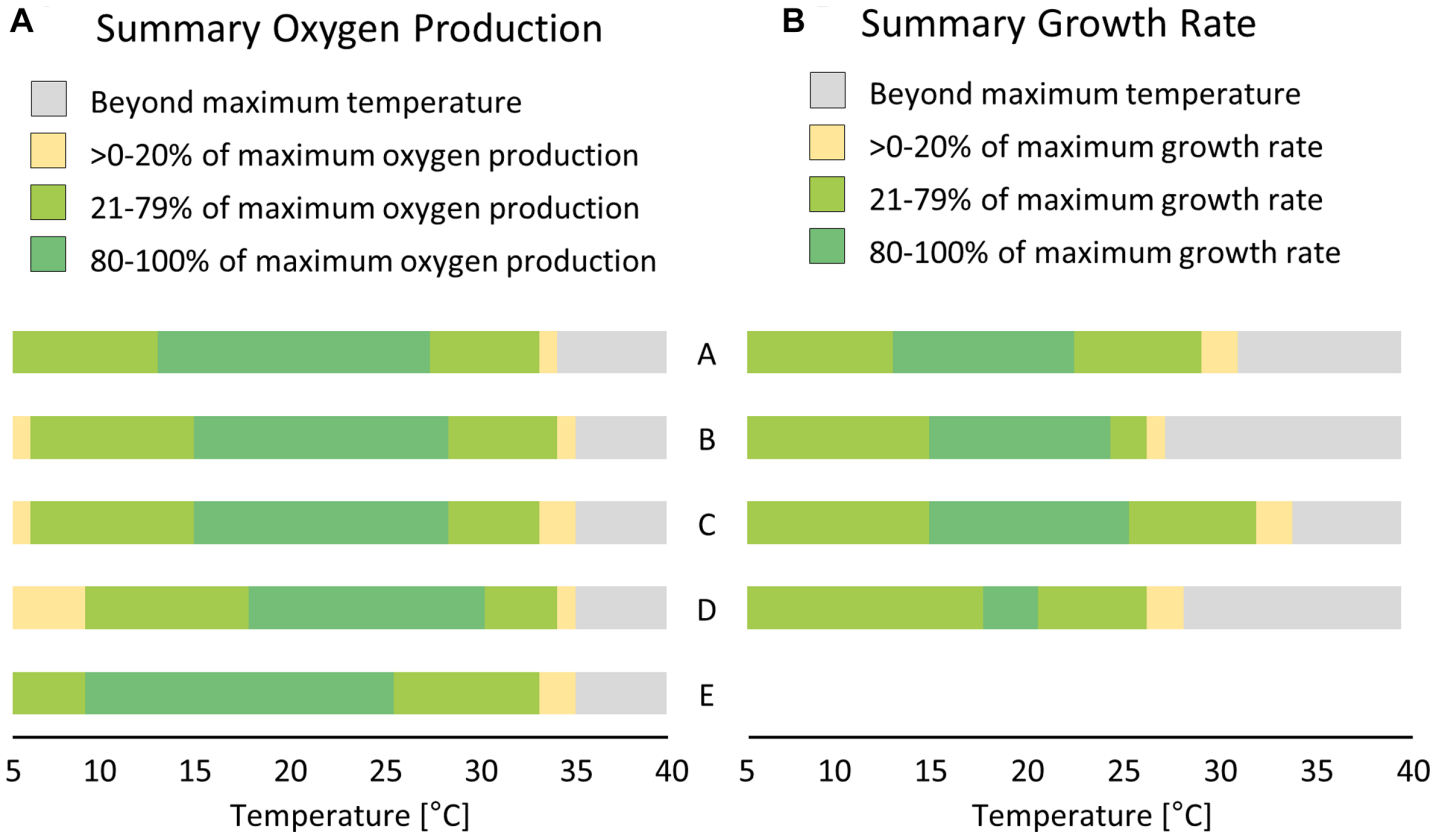
Comparison between short-term; [**(A)** few hours, measured as oxygen production] and long-term; [**(B)** few days, measured as growth rate] effects of temperature treatment on five (or four) diatom cultures isolated from biocrusts in sand dunes. Values were calculated based on the fitting results presented in Figures 6 8. A – *Achnanthes coarctata*, B – *Hantzschia abundans*, C – *Hantzschia amphioxys*, D – *Pinnularia borealis*, E – *Pinnularia intermedia.*

The photosynthetic performance of the cultures showed a broad temperature tolerance (Figure 6; Table 3). The optimal temperature range, where at least 80% of the maximum oxygen production occurred, was about 13°C (Figure 7A). *P. intermedia* had a slightly higher range of about 15°C. In addition, the temperature range (between 10°C and 25°C) of optimal oxygen production for this species was below the range of the other four cultures, which was around 13–18°C to 27–30°C. The respiration of all diatom cultures had a higher optimum temperature (30°C to 35°C) than photosynthesis.

**Table 3:** Parameters of oxygen production along a temperature gradient after fitting with Yan and Hunt (1999) model including 5% confidence interval for four diatom cultures (*n* = 4).

	*Achnanthes coarctata*	*Hantzschia abundans*	*Hantzschia amphioxys*	*Pinnularia borealis*	*Pinnularia intermedia*
NPP_max_	62.8 (53–72.7)	56.5 (46.8–66.2)	60 (46.6–73.4)	35.6 (29.4–41.9)	64.2 (53.2–75.2)
optimum temperature [°C]	20.3 (17.8–22.7)	21.6 (19.2–24)	22.1 (18.9–25.3)	24.5 (22.3–26.6)	17.1 (13.8–20.5)
maximum temperature [°C]	34.8 (33.6–36)	34.5 (33.3–35.6)	35 (33.5–36.5)	35.5 (34.5–36.5)	35.1 (33.4–36.8)

NPP_max_, maximumrates of net primary production in μmol O_2_ mg^−1^ Chl *a* h^−1^.

### 3.7 Temperature-dependent growth rate

**Figure 8:**
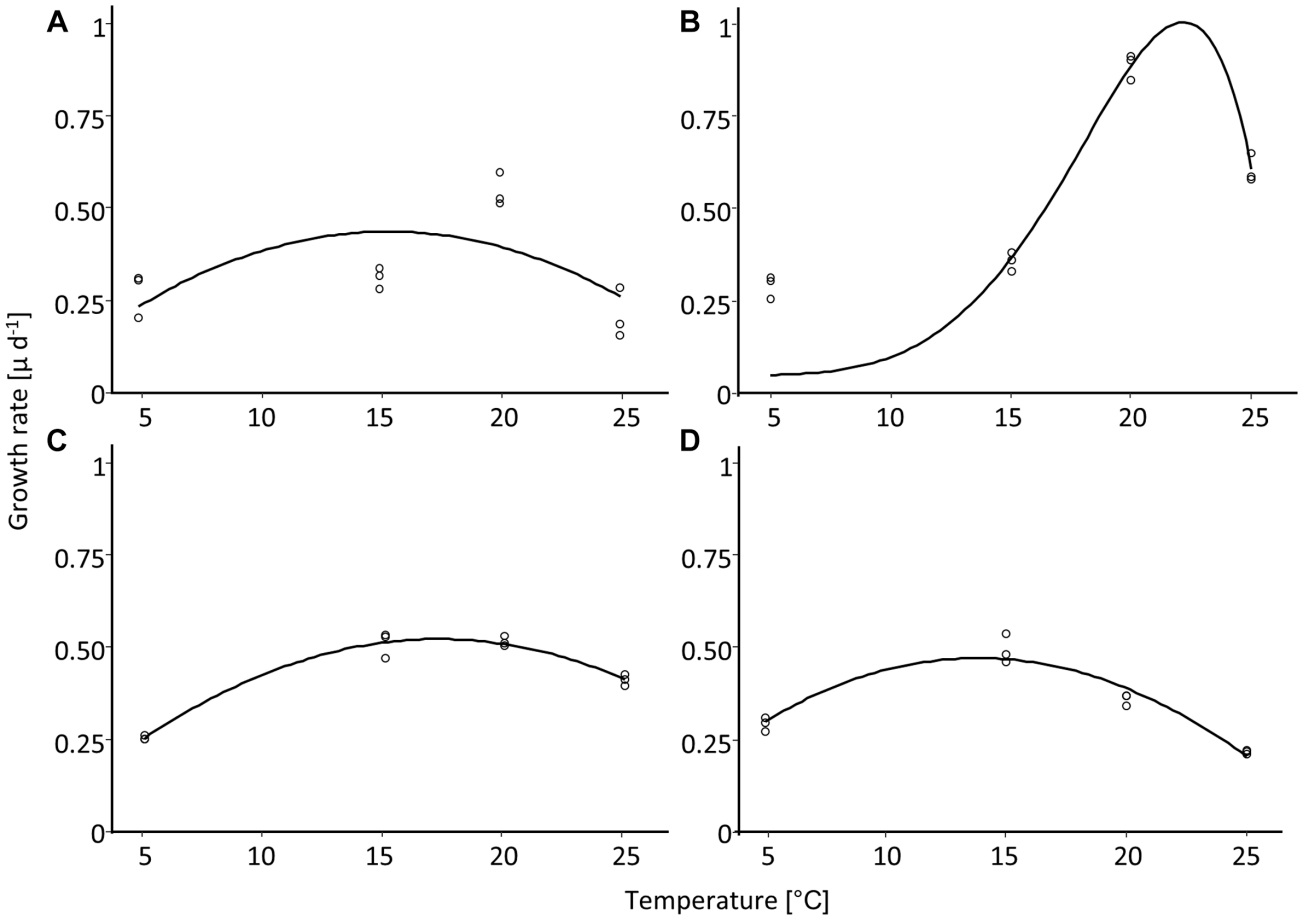
Temperature-dependent growth rate of four diatom cultures isolated from biocrusts in sand dunes. Points represent the measured values (*n* = 3), and the line is the fitting curve after Yan and Hunt (1999). **(A)** *Achnanthes coarctata*, **(B)** *Hantzschia abundans*, **(C)** *Hantzschia amphioxys*, **(D)** *Pinnularia borealis.*

The dependence of the growth rate on temperature was similar for *H. amphioxys, A. coarctata,* and *P. borealis*. These three cultures showed a similar maximum growth rate of ~0.5 μ day^−1^ (Figure 8; Table 4). *H. abundans* had a higher growth rate than the others. The maximum temperature for growth (Table 4) was highest in *H. amphioxys* (34°C), followed by *A. coarctata* (~31°C), whereas *H. abundans* and *P. borealis* reached their maximum temperature for growth already at 26°C and 28°C, respectively (Figure 7B).

**Table 4:** Parameters of growth rate along a temperature gradient after fitting with Yan and Hunt model including 5% confidence interval for four diatom cultures (no results for *Pinnularia intermedia*; *n* = 3).

	*Achnanthes coarctata*	*Hantzschia abundans*	*Hantzschia amphioxys*	*Pinnularia borealis*
maximum growth rate [μ day^−1^]	0.44 (0.31–0.57)	0.96 (0.66–1.26)	0.53 (0.51–0.55)	0.42 (0.39–0.46)
optimum temperature [°C]	15.4 (11.4–19.4)	22.2 (21.3–23)	17.2 (16.6–17.9)	13.9 (12.9–14.8)
maximum temperature [°C]	30.6 (22.2–39.0)	26.2 (25.1–27.4)	34.1 (32.1–36.2)	28 (26.7–29.2)

### 3.8 Biochemical UV protection

**Figure 9:**
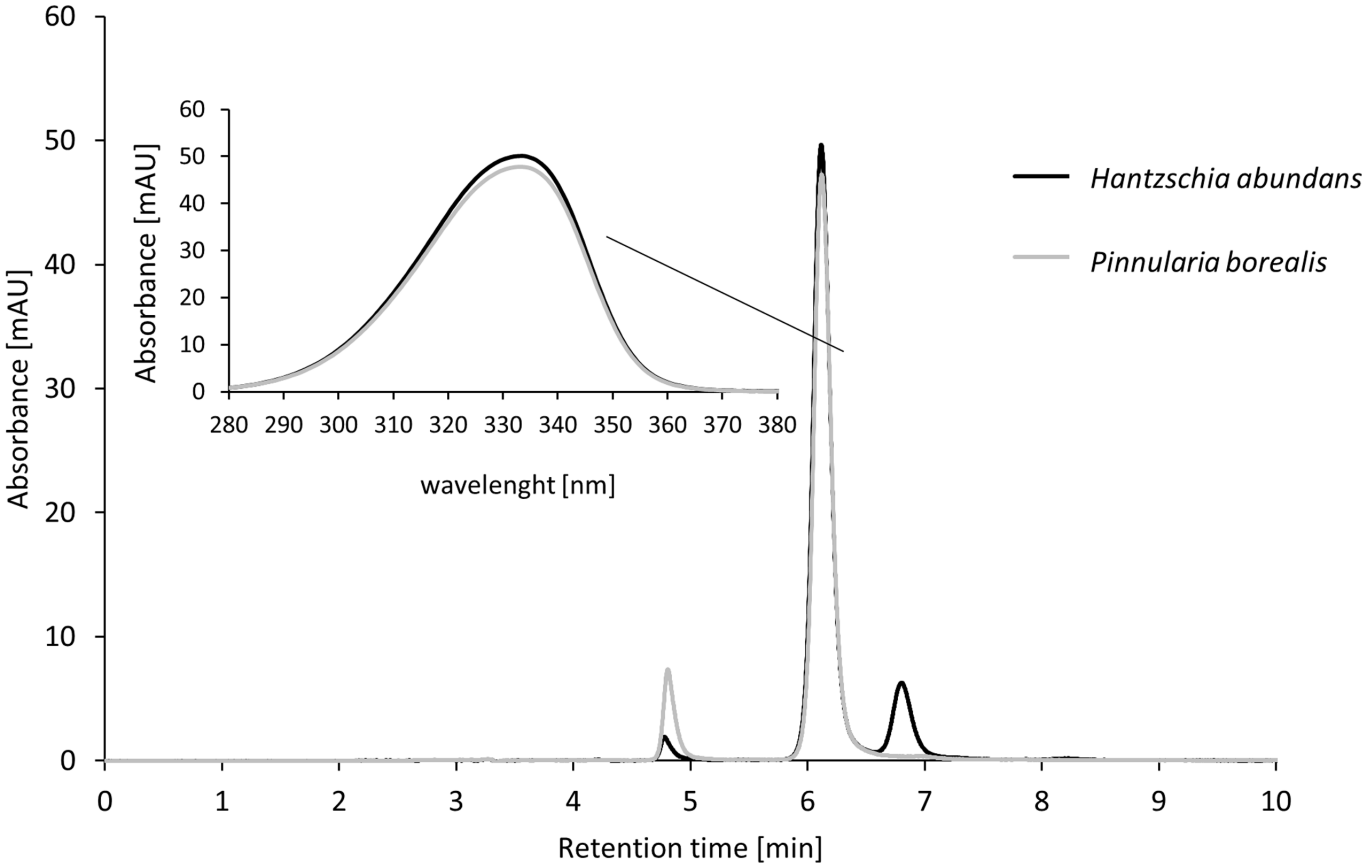
High-performance liquid chromatography (HPLC) results for mycosporine-like amino acid (MAA) extraction from terrestrial diatom cultures; two of five diatom strains contained MAAs. The insert shows the absorbance of the strongest peak (maximum absorbance at 333.5 nm).

In two of five cultures, *H. abundans* and *P. borealis*, the same mycosporine-like amino acid (MAA) was detected (Figure 9). The absorbance of this MAA was 333.5 nm wavelength and had a retention time of 6 min. This was a longer retention time than for our test standards, and therefore the MAA in these diatoms could not be identified.

## 4 Discussion

Depending on environmental conditions and external influences, biocrusts can reach different successional stages, from a thin algal crust to moss-dominated or lichen crusts (Langhans et al., 2009; Garcia-Pichel, 2023). The biocrusts observed at the little-disturbed site were mostly dominated by mosses, representing a later stage in succession (Lan et al., 2012; Garcia-Pichel, 2023). One reason for this may be the high protection status of the dunes within the national park. The dunes are protected against human trampling and biocrust can form stable layers on the dune sediment, as shown in this study. These stable biocrusts accumulated more organic material, as evidenced by the significantly higher total carbon and Chl *a* contents compared to the other two sites. Phosphorus is transported into the ecosystem mostly by wet and dry deposition (Berthold et al., 2019). Further weathering of phosphorus-containing parent rock material is an important mechanism through which phosphorus becomes available to the biocrust community in the sand dunes (Filippelli, 2002). The significantly higher phosphorus concentration at the moderately disturbed site might be explainable by a selective input of an external P source such as bird droppings, which could have influenced the measurement.

Terrestrial diatoms have long been known, but have received only slight interest from biologists. Recent studies have pointed toward a large hidden diversity within morphological species. For example, the species complexes of *P. borealis* and *H. amphioxys* have been divided into several species (Pinseel et al., 2019; Maltsev et al., 2021), which share morphological similarities but can be clearly distinguished by molecular methods. Such studies provide evidence of the gap in our current understanding of terrestrial diatoms.

In our study, we observed 28–30 diatom taxa per sampling site (a total of 47 species at the three sites combined), which is within the common range of species per sample compared to other studies (Van de Vijver et al., 2008; Norbäck Ivarsson et al., 2013; Kopalová et al., 2014; Schulz et al., 2016; Zhang et al., 2020; Fazlutdinova et al., 2021; Foets et al., 2021). However, we observed a large number of marine diatoms (for example *Achnanthes lemmermannii*, *Catenula adhaerens*, *Cocconeis peltoides*, *Navicula germanopolonica*, and *Navicula viminoides*; for ecological preferences of these diatoms see Witkowski et al., 2000; Plinski and Witkowski, 2020). These marine species probably did not live in the biocrusts, and their valves may have been blown onto the beach or transported via sea spray. This idea is supported by our observation of many partly destroyed valves. This would be uncommon if the diatoms were living and reproducing in this habitat. Therefore, it appears that only 4 to 6 species were actually abundant and alive in the biocrust samples. Among these, we found typical terrestrial diatoms such as *P. borealis* and *H. amphioxys*. *P. borealis* is reported worldwide in a variety of terrestrial habitats (Pinseel et al., 2019), even in less-favorable environments such as volcanic soils a few months after an eruption (Fazlutdinova et al., 2021). This species has outperformed other diatom species in surviving frequent and extreme freezing (−180°C) conditions (Hejduková et al., 2019). Nevertheless, it is important to keep in mind that our current understanding of *P. borealis* as a ubiquitous and highly stress-tolerant species might change. Pinseel et al. (2019) found that *P. borealis* is a complex consisting of around eight species. Similarly, *H. amphioxys*, also reported worldwide from a variety of habitats, also seems to be a complex of approximately six species. Single species within a species complex may diverge to different habitats. *A. coarctata* is also occasionally reported from terrestrial habitats, especially since it seems to live in mosses. For example, we cultured *A. coarctata*, which was reported from moss samples in Antarctica (Kopalová et al., 2014). However, we did not observe this species on our combusted slides, which may indicate that it was present in low abundance in our samples. The dominance of *P. intermedia* in the moderately disturbed area was remarkable. We also cultured this species, although we did not succeed in keeping it in culture for long. Notably, *P. intermedia* is only rarely reported and is also missing in the sequence databank. This species was observed from sand dunes (Round, 1957; Schulz et al., 2016) and mosses (Beyens, 1989), but since then has only rarely been reported in terrestrial habitats.

### 4.1 Indicator value

Freshwater diatoms have long been used as bioindicators in aquatic environments because some species are sensitive to pollution (e.g., high nutrient concentrations) and thus provide valuable information concerning water quality. The idea to use terrestrial diatoms as bioindicators is relatively new, and the criteria for assessment are not yet defined (Antonelli et al., 2017; Barragán et al., 2018). However, this approach seems promising (Wanner et al., 2020; Zhang et al., 2020; Minaoui et al., 2021). For example, a study in suburban soils around Marrakesh, Morocco, supported the idea of using diatoms for indication of high nutrient content, pH, and conductivity (Minaoui et al., 2021). Although our study showed different community patterns, possibly depending on the degree of human disturbance, there was no clear indicator species for low or high disturbance levels. In our case, maybe the high proportion of broken diatom frustules might serve as an indicator for high disturbance levels. Broken frustules indicate that these diatoms had been dead for some time and therefore the frustules became disrupted due to physical forces. These forces can be stronger under regular trampling because in such cases the sand grains would grind the silica frustules. In coastal dunes, such broken frustules could also be carried in from the sea via sea spray. We derive this assumption from our results, but further research is needed to confirm our hypothesis and to establish the proportion of broken frustules as a valid indicator value in the future.

### 4.2 Ecophysiological performance of soil diatom cultures

Most studies on terrestrial diatoms rely on estimation of morphological diversity based on valves, a well-developed method (Nagy, 2011). Nevertheless, it remains unclear if the valves represent living and reproducing diatom species or if those diatoms were dead, or if the valves were merely blown into place because valves can remain stable for several centuries and are used in paleobiology (Dreßler et al., 2011). Only a few studies on terrestrial diatoms rely on culture techniques. Culturing of terrestrial diatoms is often quite challenging due to unknown requirements and because the diatoms tend to live closely attached to soil particles. Consequently, our study is one of only a few that have examined the ecophysiological tolerance of soil diatoms. Our study showed that the diatoms can use and tolerate a wide range of irradiance intensities. Also, short-term temperature shifts could be tolerated quite well. Both factors, light intensity and temperature, can change rapidly in terrestrial habitats and over a wider range than in aquatic habitats. However, our observations of slower growth rates during several days of increased temperatures made it obvious that soil diatoms prefer relatively low to moderate temperatures (~15–20°C) and are sensitive to high temperatures (>30°C). In coastal sand dunes, the surface can reach temperatures above 40°C (Maun, 2009). It appears that the diatoms could tolerate such a shift for a short time but need a recovery period with moderate temperatures during the night. Also, other studies have reported that diatoms can tolerate a wide temperature range over the short term (Souffreau et al., 2010).

Terrestrial habitats, especially the sand dunes with their low water-holding capacity, can dry out rapidly during the day. Therefore, terrestrial organisms must deal with periodic desiccation events. Three cultures (*H. amphioxys, H. abundans,* and *A. coarctata*) reached 50% of their initial photosynthetic performance after rehydration. Only the two *Pinnularia* species (*P. borealis* and *P. intermedia*) failed to recover well after the desiccation event. This is somewhat surprising because both *Pinnularia* species are true terrestrial species and can live outside a moss carpet. It might be that our experimental setup was harsh, with a relative humidity below 25%, which does not naturally occur in coastal dunes in Germany. Additionally, the *P. intermedia* cells were smaller than average, which indicates that the culture did not reach its full potential. In general, pennate diatoms, such as those in our five cultures, are mobile and can avoid unfavorable conditions or migrate in a positive phototactic direction (e.g., moving vertically in sediments) under natural conditions (Consalvey et al., 2004; Poulsen et al., 2022). Such a protective vertical movement was not possible for the diatoms during the desiccation experiment. A study on aquatic and terrestrial diatoms also indicated a low desiccation tolerance for diatoms in general when only air-dried (Souffreau et al., 2010). The exceptions in this study were *P. borealis* and *H. amphioxys*, which were able to grow after drying in air for 10 min.

### 4.3 Mycosporine-like amino acids from diatoms

Mycosporine-like amino acids are frequently reported from marine or brackish water and are mostly attributed to dinophytes and bacillariophytes (Jeffrey et al., 1999). Interestingly, it was suggested that MAAs might be stored in the silica frustules, which would substantially enhance their stability and prevent harmful UV light from penetrating the outer cell layer (Ingalls et al., 2010). We did not test for the location of the MAAs, but this seems to be an interesting strategy, which could potentially also be used by terrestrial diatoms. Studies on marine diatom cultures have reported seven MAAs as the most common: Shinorine, Porpyhra-334, Mycosporine-2-glycine, Palythine, Palythene, Mycosporine-glycine, and Mycosporine-glycine-valine (Helbling et al., 1996; Moisan and Mitchell, 2001). Of these frequently present diatom MAAs, we most likely detected Mycosporine-2-glycine, with an absorbance at 334 nm. This assumption is supported by the retention time for Mycosporine-2-glycine in a methanol/acid mobile phase in HPLC, which is a few seconds longer than for Porphyra-334. In our study, the MAA from the terrestrial diatoms had a slightly longer retention time in the same phase than the Porphyra-334 standard.

Our study contributes the first record of MAAs in terrestrial diatoms. Terrestrial habitats are characterized by high UV radiation compared to aquatic habitats, especially in summer under a clear sky. Terrestrial diatoms are typically pennate with one or two raphes, which allow the diatoms to move, for example, in marine sediments. Therefore, we hypothesize that terrestrial diatoms could move in deeper sand layers to avoid harmful radiation. This would incur a certain cost: although the diatoms escape the harmful UV radiation, they are less exposed to photosynthetic active radiation. As all the cultures that we tested showed no photoinhibition, protection from harmful UV radiation seems to be a useful strategy to exploit the high radiation on the sand surface for efficient photosynthesis without being harmed by UV radiation.

## 5 Conclusion

In this study, we evaluated the ecophysiological characteristics of terrestrial diatoms from sand dunes. Although terrestrial diatoms have been known for a long time, only a few studies have investigated their functioning despite their abundance and wide distribution. Our study is one of only a few to provide an insight into the ecology of terrestrial diatoms based on cultures, which is a necessary basis for understanding their ecology and distribution. We observed a wide temperature tolerance of all diatom cultures concerning primary production, but long-term exposure to different temperatures resulted in a lower temperature tolerance concerning growth rates. Desiccation tolerance differed among the cultures, with some showing a high recovery rate after harsh desiccating conditions and others with only low recovery rates. Although terrestrial diatoms appear to be sensitive to warmer and drier summer conditions, the biocrust microecosystem including a moss cushion may reduce the environmental stress for these diatoms.

## Data availability statement

The original contributions presented in the study are publicly available. This data can be found here: NCBI, accession numbers OR387857-OR387860.

## Author contributions

KG: Formal analysis, Investigation, Conceptualization, Data curation, Funding acquisition, Project administration, Supervision, Visualization, Writing – original draft. SK: Formal analysis, Investigation, Writing – review & editing. NP: Data curation, Formal analysis, Methodology, Visualization, Writing – review & editing. MD: Data curation, Formal analysis, Methodology, Visualization, Writing – review & editing.
